# Treatment of Established Status Epilepticus

**DOI:** 10.3390/jcm5050049

**Published:** 2016-04-25

**Authors:** Jessica J. Falco-Walter, Thomas Bleck

**Affiliations:** Department of Neurology, Rush University Medical Center, 1725 West Harrison Street, Suite 885, Chicago, IL 60612, USA; tbleck@rush.edu

**Keywords:** status epilepticus, established status epilepticus, treatment, antiseizure, phenytoin, levetiracetam, valproic acid, phenobarbital, lacosamide

## Abstract

Status epilepticus is the most severe form of epilepsy, with a high mortality rate and high health care costs. Status epilepticus is divided into four stages: early, established, refractory, and super-refractory. While initial treatment with benzodiazepines has become standard of care for early status epilepticus, treatment after benzodiazepine failure (established status epilepticus (ESE)) is incompletely studied. Effective treatment of ESE is critical as morbidity and mortality increases dramatically the longer convulsive status epilepticus persists. Phenytoin/fosphenytoin, valproic acid, levetiracetam, phenobarbital, and lacosamide are the most frequently prescribed antiseizure medications for treatment of ESE. To date there are no class 1 data to support pharmacologic recommendations of one agent over another. We review each of these medications, their pharmacology, the scientific evidence in support and against each in the available literature, adverse effects and safety profiles, dosing recommendations, and limitations of the available evidence. We also discuss future directions including the established status epilepticus treatment trial (ESETT). Substantial further research is urgently needed to identify these patients (particularly those with non-convulsive status epilepticus), elucidate the most efficacious antiseizure treatment with head-to-head randomized prospective trials, and determine whether this differs for convulsive *vs.* non-convulsive ESE.

## 1. Introduction

Status epilepticus is the most severe and most deadly form of epilepsy. Its annual incidence is 10–41 per 100,000 people. About 5% of adults and 10%–25% of children with epilepsy will have status epilepticus at least once during the course of their lives [1]. The mortality rates in status epilepticus are high: 24%–26% in adults and 3%–6% in children [2], and an overall mortality rate of about 20% [3].

**Figure 1:**
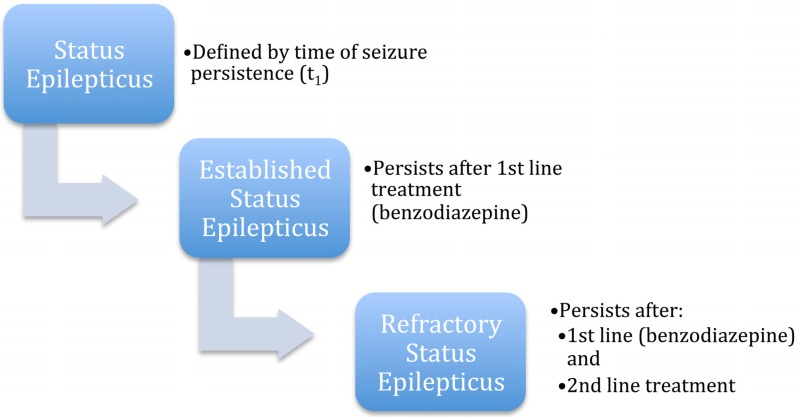
Timeline of the progression of status epilepticus.

Status epilepticus had previously been defined as continuous seizure activity lasting greater than five min. Some studies used 10 min and other studies used 30 min as a cut-off, depending upon whether there were convulsions or not. Recently the International League Against Epilepsy (ILAE) redefined status epilepticus as ongoing seizure activity due to failure of mechanisms responsible for seizure termination or initiation of mechanisms provoking ongoing seizures causing prolonged seizures after timepoint t_1_, and which can have long-term consequences after timepoint t_2_, with t_1_ and t_2_ being 5 min and 30 min, respectively for convulsive status epilepticus, 10 min and 60 min for focal status epilepticus with impaired consciousness, and 10–15 min and unknown for absence status epilepticus (Table 1) [4]. Established status epilepticus is defined as status epilepticus that persists after treatment with a benzodiazepine. Refractory status epilepticus (RSE) occurs when status epilepticus fails to abort after a first line (usually a benzodiazepine) and a second-line antiseizure medication have been given. Time is not part of the definitions for either established or refractory status epilepticus, which are based solely the medications given and persistence of seizures (Figure 1).

The longer status epilepticus persists, the less likely it will resolve spontaneously and the higher the mortality. The higher mortality rates are frequently thought to be due to the underlying cause of the seizures. Prolonged status epilepticus is most often associated with severe brain dysfunction from encephalitis, massive stroke, or large brain tumors in adults [5]. In children, status epilepticus is due to: fever, low anticonvulsant levels, electrolyte imbalances, inborn errors of metabolism, ingestions, CNS infections, bacteremia, and various neuroimaging abnormalities (cortical malformations, trauma, stroke/hemorrhage, tumors, arteriovenous malformations, hydrocephalus) [6].

Our aim in this review is to discuss the pharmacologic treatment of established status epilepticus (ESE) and the current evidence that exists for these treatments. Data sources utilized include MEDLINE and back-tracking of references in pertinent studies. The following search terms were queried: “established status epilepticus”, “benzodiazepine AND status epilepticus”, “treatment AND status epilepticus”, “phenytoin *vs.* phenobarbital in status epilepticus”, “phenytoin *vs.* valproic acid in status epilepticus”, “phenytoin *vs.* levetiracetam in status epilepticus”, “phenytoin *vs.* lacosamide in status epilepticus”, “lacosamide AND status epilepticus”, “levetiracetam AND status epilepticus”, “valproic acid AND status epilepticus”, “randomized controlled AND status epilepticus.” The abstracts from the resulting studies were reviewed; studies in which benzodiazepines were not administered first were excluded. References in articles that were particularly pertinent were retrieved and reviewed as well.

## 2. Causes of Ongoing Status Epilepticus

The duration of status epilepticus prior to treatment and the underlying etiology are the most important factors governing whether drugs will stop the seizure activity [7]. When seizures persist for longer periods of time, resistance to antiseizure medications occurs. Some medications are thought to lose their efficacy due to the ongoing seizure activity causing internalization, and therefore loss, of GABA_A_ receptors from the synaptic membrane. This leads to less inhibition, and thus ongoing seizures. Additionally, seizure activity causes an increase in the number of excitatory receptors (NMDA and AMPA) in the synaptic membrane. These changes explain why medications that increase GABA become less effective, while those that work on NMDA and AMPA receptors are unaffected. Other mechanisms for drug failure in prolonged status are less well understood. Theories exist about seizures causing an increase in cytokines and other proinflammatory markers, as well as of upregulation of drug-efflux transporters such as P-glycoprotein. To date studies have not conclusively shown that either of these mechanisms changes the efficacy of antiseizure medications [8].

## 3. Pharmacologic Treatment

After a patient has received a benzodiazepine, and their seizures persist, phenytoin or fosphenytoin has been the standard treatment, with fosphenytoin favored in the United States. In Europe it is more common for other antiseizure medications to be given after benzodiazepine failure. Some of the reason for this difference may be due to the higher cost of fosphenytoin in the past, though now phenytoin and fosphenytoin are similar in price. Phenytoin/fosphenytoin has been favored, not because of its proven superiority in treating seizures or status epilepticus, but more because of familiarity with its use as well as its long half-life. More recently it has been proposed that valproic acid, levetiracetam, phenobarbital or phenytoin/fosphenytoin should all be considered for use as the second antiseizure medication following a benzodiazepine [9]. An older clinical practice guideline from the European Federation of Neurological Sciences (EFNS) recommended phenytoin be the standard first-line agent for ESE [10], but more recent clinical practice guidelines from the Neurocritical Care Society and the American Epilepsy Society are ambivalent as to the best agent for ESE [11,12]. There are few controlled, randomized, blinded clinical trials evaluating the different medications available for the treatment of established status epilepticus and no class 1, head-to-head, blinded comparisons of these medications for the treatment of ESE.

### 3.1. Phenytoin/Fosphenytoin

Fosphenytoin and phenytoin’s primary mechanism of action is inhibition of sodium channels. Phenytoin is insoluble in water, requiring an alkaline solvent to prevent precipitation. This alkalinity can cause local irritation, thrombophlebitis, compartment syndrome, purple glove syndrome, and tissue necrosis with extravasation [13]. Fosphenytoin is water soluble, enabling it to be given intramuscularly (IM), which phenytoin cannot be. Fosphenytoin has generally been preferred to phenytoin given its better side-effect profile. It can be loaded faster intravenously (IV), has a lower risk of causing arrhythmias, hypotension, and local adverse reactions if extravasation occurs [14]. These benefits are less than was initially claimed as even fosphenytoin causes arrhythmias and hypotension, and the only significant benefit appears to be a lower incidence of purple glove syndrome. While fosphenytoin can be given faster IV, it has the same time to effect on seizures, as it must be converted to phenytoin, which takes about 15 min.

The efficacy of phenytoin for ESE was shown to be 43.1% in a large randomized study for convulsive status epilepticus in adults [15]. A retrospective study in children also demonstrated the efficacy of phenytoin after diazepam failure [16]. A recent meta-analysis pooled data from 22 studies and compared the efficacy of several antiseizure drugs for ESE. Using data from eight studies, the authors noted a mean efficacy of 50.2% for phenytoin in aborting ESE. When compared to the efficacy they found for phenobarbital, valproic acid, and levetiracetam, the efficacy of phenytoin was much less [17]. Thus, while phenytoin is efficacious, it appears less so than some of the newer antiseizure drugs. Additionally, it has a worse side-effect profile and more drug-drug interactions due to it being a cytochrome P450 inducer. However, it is significantly cheaper than many of the newer antiseizure drugs, which is an important consideration in developing countries and as escalating medical costs become a bigger concern.

Phenytoin and fosphenytoin are dosed by weight, 15–20 mg/kg as an initial loading dose, after which an additional 5–10 mg/kg may be given if the initial dose is insufficient. Two hours after the loading dose a level should be checked, with the goal being a level of 15–20 μg/mL. Giving a loading dose of 1 gm for everyone is not appropriate. Hypotension may still be seen with fosphenytoin; thus, blood pressure and electrocardiographic (EKG) monitoring is needed for these infusions [11,12].

### 3.2. Valproic Acid

Valproic acid, while newer than phenytoin, has been approved by the FDA since the 1960s and is the next best studied. It has multiple mechanisms of action, which include multiple actions on GABA, NMDA-receptor antagonism, and histone deacetylase inhibition [18].

There are several trials that have compared valproic acid to other antiseizure drugs. Valproic acid has been shown to be as effective as, or more effective than phenytoin in two studies, by Gilad *et al.* and Misra *et al.* respectively. Seizures were aborted in 66%–88% of patients with status epilepticus or acute repetitive seizures when they were given valproic acid in these studies. Notably, in both of these studies benzodiazepines were not administered first, as is currently recommended, and phenytoin was likely disadvantaged by its time to onset [19,20]. Agarwal *et al.* compared IV phenytoin to IV valproic acid for ESE refractory to IV diazepam and found that they were equivalent in stopping ESE. The complication rates (hypotension and respiratory depression) were slightly higher in the phenytoin group as compared to the valproic acid group, which was significant [21]. Malamiri *et al.* compared valproic acid to phenobarbital in children who failed a single dose of IV diazepam (0.2 mg/kg) and found no significant difference in achieving seizure control after 20 min, but a significant difference was found for seizure recurrence within 24 h. At 24 h the patients who received phenobarbital boluses and maintenance were more likely to have recurrence of seizures then those who received valproic acid boluses and maintenance doses (37% without recurrence with phenobarbital *vs.* 77% with valproic acid) [22]. Chen *et al.* compared valproic acid to diazepam infusion after initial diazepam administration failed to stop seizures in adults with established generalized convulsive status epilepticus. Both medications were found to be equally efficacious, at 50% and 56% response rates, respectively. Notably, there were more complications (hypotension, need for artificial ventilation, and vasopressor support) for the patients who received the midazolam infusion [7]. Finally, the meta-analysis discussed above by Yasiry and Shorvon pooled data from eight studies on valproic acid, and showed an efficacy of 75.7% in aborting ESE. This was the highest efficacy of any of the medications reviewed [17]. From reviewing the above studies valproic acid is clearly at least as effective as phenytoin in aborting ESE.

Of note, valproic acid is a cytochrome P450 inhibitor, thus interacting with many other medications, similar to phenytoin. While it is less cardiotoxic than phenytoin, it is not free of adverse effects, most notably hepatotoxicity, thrombocytopenia, hyperammonemia, and acute hemorrhagic pancreatitis [13]. This agent is particularly problematic in patients with inborn errors of metabolism, such as ornithine carbamyltransferase deficiency.

Valproic acid is administered as 20–40 mg/kg IV over 10 min, and an additional 20 mg/kg can be given over 5 min if the patient is still seizing. The goal blood level is 100 μg/mL, and can be drawn immediately after the loading dose has been administered. Maximum dose of 3 gm. Cardiac complications are not an issue with valproic acid [11,12].

### 3.3. Phenobarbital

Phenobarbital aborts seizures primarily by enhancing GABAergic inhibition and secondarily by inhibiting sodium currents. It has been more extensively used in developing countries and in the pediatric population, though there are surprisingly few head-to-head studies looking at its efficacy.

As discussed above, Malamiri *et al.* compared IV valproic acid to IV phenobarbital in children after diazepam failure and found no difference initially in efficacy, but a higher recurrence of seizures in the phenobarbital group at 24 h [22]. Yasiry and Shorvon found an efficacy of 73.6% in their meta-analysis, which included just two papers, looking at 42 patients in total [17].

In developing countries, where cost is paramount, phenobarbital use is more common. Being a cytochrome P450 inducer, it interacts with many medications and has a worse side effect profile, with a propensity to cause hypotension, sedation, and respiratory depression, particularly with rapid infusion [13]

Phenobarbital should be given as a bolus of 10–20 mg/kg IV at a rate of 50–100 mg/minute, up to a total amount of 700 mg in seven min. Patients must have their respiration and blood pressure monitored while they are receiving the bolus [11].

### 3.4. Levetiracetam

Levetiracetam is a pyrrolidone derivative and piracetam analog. Its precise mechanism of action is unknown; it binds to synaptic vesicle protein 2A (SV2A) [18], but how this may affect seizures is presently unclear.

While there are not any head-to-head, prospective trials of levetiracetam, it appears to be as effective as valproic acid and other antiseizure drugs in the data that currently exist for ESE [23]. Knake *et al.* performed a retrospective study in which they found that levetiracetam controlled 16/18 patients in ESE [24]. Yasiry and Shorvon found an efficacy of 68.5% in their meta-analysis of eight studies. This was less than the efficacy of phenobarbital and valproic acid, but greater than that of phenytoin [17]. However, Alvarez *et al.* questioned whether levetiracetam is as efficacious for ESE. In their retrospective comparison of levetiracetam, valproic acid, and phenytoin, each medication failed to abort ESE in 48.3%, 25.4%, and 41.4% of patients, respectively. Thus, levetiracetam was significantly less efficacious than valproic acid and phenytoin [25].

Unlike the medications discussed above, levetiracetam has a significantly better adverse effect, interaction, and safety profile. It does not affect the cytochrome P450 enzymes. Its IV formulation is slightly more expensive than phenytoin.

Levetiracetam is given as 2.5 gm over 5 min or 1–4 gm IV over 15 min. A maximum of 4.5 gm can be given. Levetiracetam has the advantage of not causing many adverse reactions and also not interacting with other medications. It does accumulate in patients with renal dysfunction, however, and maintenance doses must be reduced in this setting [11,12].

### 3.5. Lacosamide

Lacosamide aborts seizures by selectively enhancing the slow inactivation of voltage-gated sodium channels [18]. While it only came to market in 2008, its high rate of off-label use for refractory status epilepticus is likely due to its availability in the IV form, which has been available since 2009.

There are no studies specifically looking at lacosamide in ESE, and only a handful looking at its efficacy in SE (19 studies, 10 single case-reports and 9 case-series) looking at a total of 136 patients. These studies showed an overall success of aborting status of 56%. The most common side effects were mild sedation and hypotension [13,26]. Yasiry and Shorvon were able to obtain additional data from the authors of the studies published, which resulted in only four patients qualifying as receiving lacosamide for ESE. Thus they concluded that there was insufficient data at this time [17]. A randomized trial of lacosamide and fosphenytoin (TRENdS) for NCSE is still being analyzed, but it was stopped prematurely due to difficulty recruiting patients. This study may elucidate where lacosamide fits into treatment, but it will likely be under-powered [27].

The most commonly used bolus dose is 400 mg IV, followed by a daily dose of 200–400 mg given in divided doses [11].

## 4. Future Directions

While the medications discussed above all appear to have some evidence for halting status epilepticus that is resistant to benzodiazepines alone, there are no class 1, head-to-head, blinded comparisons of these medications for the treatment of ESE. To address this lack of evidence, the Established Status Epilepticus Treatment Trial (ESETT), is currently enrolling patients. This study will compare the efficacy of fosphenytoin, levetiracetam, and valproic acid. Patients who are greater than two years of age, with witnessed generalized tonic–clonic activity that is ongoing in the emergency room for over five min and who have failed to respond to benzodiazepines will be enrolled. The benzodiazepine may have been given in divided doses and may be given prior to arrival or in the hospital. The benzodiazepine doses acceptable as first line are: diazepam 10 mg IV, lorazepam 4 mg IV, midazolam 10 mg IV or IM if they are over 40 kg. For patients less than 40 kg they must have received lorazepam 0.1 mg/kg IV, midazolam 0.3 mg/kg IV or IM. Patients meeting the inclusion criteria will be randomized to one of three arms: fosphenytoin at 20 mg/kg, levetiracetam at 60 mg/kg or valproic acid at 40 mg/kg. The medications will be formulated so they will all infuse over 10 min. Patients will be assessed for seizure resolution as well as for hypotension and arrhythmias. ESETT plans to enroll up to 795 patients, but interim analyses will be performed at 400, 500, 600 and 700 patients, to assess for a higher success rate or of a failure of any of the medications [28,29,30].

Additionally, the Emergency Treatment with Levetiracetam or Phenytoin in status epilepticus in Chidren (EcLiPSE) Trial is currently enrolling patients. This study will compare the efficacy of levetiracetam and phenytoin in children. This study is being conducted at multiple centers in the United Kingdom. Patients must be 6 months to 18 years of age, with convulsive status epilepticus (generalized tonic–clonic, generalized clonic, or focal clonic) that is ongoing and has failed to respond to first line treatment. First line treatment is defined as a benzodiazepine of any sort by any route or rectal paraldehyde. Patients meeting the inclusion criteria will receive either levetiracetam 40 mg/kg (up to a maximum of 2500 mg) IV over 5 min or phenytoin 20 mg/kg (up to a maximum of 2000 mg) IV over 20–40 min (rate dependent upon dosage). Patients will be assessed for time to visible seizure cessation as well as whether an additional agent was required, if intubation or ICU admission was needed, and for any complications. They plan to enroll 340 patients. The study began enrolling patients in 2014 and is expected to finish in March of 2019 [31,32].

Until these trials are complete, the studies discussed above provide the best evidence for management of ESE. While phenobarbital was proposed by Betjemann and Lowenstein as a second line agent after benzodiazepine failure, ESETT and EcLiPSE will not address the efficacy of phenobarbital. Lacosamide has also been proposed as a second line agent, but there are minimal data on its efficacy in ESE and it also will not be included in ESETT or EcLiPSE.

The vast majority of the patients included in the studies discussed above had convulsive status epilepticus. In some of the studies patients with non-convulsive status epilepticus (NCSE) were specifically excluded, and in other studies they were included, but their numbers were small and the studies were not powered to make conclusions about this subset. The percentage of individuals with NCSE in intensive care units is estimated to be up to 20%, but there is little data about the best treatment for these individuals. While there is convincing evidence that ongoing generalized convulsive seizures is damaging, particularly after 30 min, there is little evidence to indicate that non-convulsive status epilepticus causes similar damage. There is even some evidence to indicate that being overly aggressive with more sedating AEDs is detrimental, particularly in the elderly. As a result, many interventionalists feel a less aggressive treatment approach to non-convulsive status epilepticus is appropriate [18]. Whether the subset of NCSE should be treated differently in regard to ESE is unclear, and further studies will need to be completed.

## 5. Conclusions

The current evidence supports the use of valproic acid, levetiracetam, phenobarbital, or phenytoin all as treatments for ESE. There is insufficient data to support the use of lacosamide at this time. Valproic acid appears to be slightly more efficacious in a handful of the studies discussed, but until ESETT is completed any of these medications could arguably be considered equivalent. Additionally, given the adverse effect profile of each medication, for a given patient, one agent may be preferable. Overall there is a definite need for prospective controlled randomized trials for ESE.

## Figures and Tables

**Table 1:** Definitions of Status Epilepticus.

**ILAE Definitions of Status Epilepticus**		
	Time after which if seizures do not terminate patient is considered in status epilepticus (t_1_)	Time after which ongoing seizures have long term consequences (t_2_)
Convulsive status epilepticus	5 min	30 min
Focal status epilepticus with impaired consciousness	10 min	60 min
Absence status epilepticus	10–15 min	unknown
**Other Definitions of Status Epilepticus**		
Established statusepilepticus	Status epilepticus that persists after treatment with a benzodiazepine (1st line treatment)	
Refractory status epilepticus	Status epilepticus that persists after a 1st line agent (benzodiazepine) and 2nd lines	
	agent (additional agent such as levetiracetam, phenytoin, valproic acid) have failed	
